# Development of a Pig Mammary Epithelial Cell Culture Model as a Non-Clinical Tool for Studying Epithelial Barrier—A Contribution from the IMI-ConcePTION Project

**DOI:** 10.3390/ani11072012

**Published:** 2021-07-05

**Authors:** Chiara Bernardini, Debora La Mantia, Roberta Salaroli, Augusta Zannoni, Nina Nauwelaerts, Neel Deferm, Domenico Ventrella, Maria Laura Bacci, Giuseppe Sarli, Michele Bouisset-Leonard, Pieter Annaert, Monica Forni

**Affiliations:** 1 Department of Veterinary Medical Sciences, University of Bologna, Ozzano dell’Emilia, 40064 Bologna, Italy; chiara.bernardini5@unibo.it (C.B.); debora.lamantia2@unibo.it (D.L.M.); roberta.salaroli@unibo.it (R.S.); domenico.ventrella2@unibo.it (D.V.); marialaura.bacci@unibo.it (M.L.B.); giuseppe.sarli@unibo.it (G.S.); monica.forni@unibo.it (M.F.); 2 Health Sciences and Technologies-Interdepartmental Center for Industrial Research (CIRI-SDV), Alma Mater Studiorum—University of Bologna, 40126 Bologna, Italy; 3 KU Leuven Drug Delivery and Disposition Lab, Department of Pharmaceutical and Pharmacological Sciences, O&N II Herestraat, 49 3000 Leuven, Belgium; nina.nauwelaerts@kuleuven.be (N.N.); neel.deferm@kuleuven.be (N.D.); pieter.annaert@kuleuven.be (P.A.); 4 Novartis Pharma AG, Novartis Institutes for BioMedical Research, Werk Klybeck Postfach, CH-4002 Basel, Switzerland; michele.bouisset-leonard@novartis.com

**Keywords:** lactation, breastfeeding, mammary gland, pig model, mammary epithelial cells, barrier function

## Abstract

**Simple Summary:**

The information about the risks related to the use of medication during breastfeeding is lacking for most commonly used drugs. The ConcePTION project aims to fill this gap using multiple approaches. Within the project, the pig has been selected as the most appropriate in vivo animal model. In agreement with the application of the “3Rs” principle (Replacement, Reduction and Refinement) and international legislations, the present paper reports the establishment of cellular lines of porcine mammary epithelial cells as a valid tool to study the mammary epithelial barrier function in vitro.

**Abstract:**

The ConcePTION project aims at generating further knowledge about the risks related to the use of medication during breastfeeding, as this information is lacking for most commonly used drugs. Taking into consideration multiple aspects, the pig model has been considered by the consortium as the most appropriate choice. The present research was planned to develop an efficient method for the isolation and culture of porcine Mammary Epithelial Cells (pMECs) to study the mammary epithelial barrier in vitro. Mammary gland tissues were collected at a local slaughterhouse, dissociated and the selected cellular population was cultured, expanded and characterized by morphology, cell cycle analysis and immunophenotyping. Their ability to create a barrier was tested by TEER measurement and sodium fluorescein transport activity. Expression of 84 genes related to drug transporters was evaluated by a PCR array. Our results show that primary cells express epithelial cell markers: CKs, CK18, E-Cad and tight junctions molecules ZO-1 and OCL. All the three pMEC cellular lines were able to create a tight barrier, although with different strengths and kinetics, and express the main ABC and SLC drug transporters. In conclusion, in the present paper we have reported an efficient method to obtain primary pMEC lines to study epithelial barrier function in the pig model.

## 1. Introduction

ConcePTION is a European IMI (Innovative Medicines Initiative) project, approved in April 2019, with the aim of generating accurate knowledge about the use of medication during pregnancy and breastfeeding. At the present moment, information on the safety of maternal medication during breastfeeding is lacking for most drugs [1].

The Work Package 3 (WP3) of ConcePTION aims to generate a non-clinical testing platform to establish drug transfer into human breast milk, and subsequent neonatal exposure. To achieve this goal, different approaches will be followed including in vivo and in vitro studies with animal models. Animal models have been used successfully in the past to predict the safety of maternal medication during breastfeeding, but caution is necessary due to species specific differences (e.g., in transporters, enzymes and milk composition) [2]. Among different animal species considered, the pig has been chosen as the most fit for purpose model within the ConcePTION project [3,4]. In fact, even though several macroscopic differences exist between pig and human mammary gland anatomy, many similitudes have been reported at the molecular level. Within the complex structure of a functional mammary gland, mammary epithelial cells (MECs) are engaged not only in the secretory function but also in xenobiotic transfer from mother to newborn [5,6]. Given that, in vitro studies with MECs have been proposed as a helpful approach to determine the possibility of transepithelial drug transport and rate [7,8,9,10]. Therefore, given the importance of the model, MECs were isolated from different species and by different methods, with variable results in terms of both quality and quantity [11]. In 2001, Kumura et al. [12] first described the isolation of porcine mammary epithelial cell (pMECs) from mammary tissue of a 180-day-old sow using a floating collagen culture system. Then, Zheng & He [13] isolated pMECs from a three-year old lactating Guanzhong pig utilizing the technique of explant migration. Sun et al. [14] and Dahanayaka et al. [15] reported the isolation of pMECs by associating manual mechanical and enzymatic dissociation. However, a fully-characterized pMECs in vitro model to study the mammary epithelial barrier is still lacking. The present research was planned to develop an efficient method of pMECs isolation from tissue collected from slaughterhouses, aiming to evaluate the external validity of the in vivo porcine animal model and determine its accuracy as a solid translational model for the study of mammary epithelial barrier in the full respect of the Reduction principles [16].

## 2. Materials and Methods

### 2.1. Chemicals and Reagents

Dulbecco Phosphate Buffered Saline (DPBS), Phosphate Buffered Saline (PBS), DPBS without calcium and magnesium, Fetal Bovine Serum (FBS), trypsin-EDTA, Antibiotic-Antimycotic (anti-anti) gentamicin, recombinant human epidermal growth factor (hEGF), Dulbecco’s Modified Eagle Medium: Nutrient Mixture F-12 (DMEM/F12), RNaseA/T1 and glucose were purchased from Thermo Fisher Scientific (Waltham, MA, USA). Propidium iodide (PI) and Multi Tissue Dissociation Kit1 were purchased from Miltenyi Biotec (Bergisch Gladbach, Germany). TRI Reagent was purchased from Molecular Research Center In, OH, USA and NucleoSpin RNA II kit from Macherey-Nagel GmbH & Co. KG, Düren, Germany. RT2 First Stand Kit, RT2 SYBR Green qPCR Mastermix and RT² Profiler™ PCR Array Pig Drug Transporters (Cat. No. PASS-070ZD) were purchased from Qiagen Hilden, Germany. Dimethyl sulfoxide (DMSO), Diaminobenzidine and Fluoroshield^TM^ with DAPI histology mounting medium were purchased from Sigma-Aldrich (St. Louis, MO, USA). Betadine 10% cutaneous solution was purchased from Meda Pharma Spa (Milan, Italy). EZ-PCR Mycoplasma Detection kit was purchased from Biological Industries (Kibbutz Beit-Haemek, Israel). Avidin-biotin-peroxidase kit, ABC Kit Elite, was purchased from Vector, Burlingame, CA, USA). Tissue embedding medium OCT was obtained from Sakura (Finetek, Torrance, CA USA). Sodium fluorescein was purchased from Siegfield Zofingen, (Switzerland). All plastic supports for cell culture were purchased from Corning-Beckton-Dickinson (Franklin Lakes, NJ, USA). Hanks Balanced Salt Solution (HBSS) was purchased from Lonza (Walkersville, MD USA) 2-[4-(2-hydroxyethyl)piperazin-1-yl] ethanesulfonic acid (HEPES) was purchased from VWR International (Milan, Italy). Fluorescein sodium was purchased from Siegfield (Zofingen Switzerland). The culture medium used for cell isolation, named the Isolation Medium (Im), was the Epithelial Mammary Cell Medium supplemented with 0.004 mL/mL Bovine Pituitary Extract (BPE), 10 ng/mL hEGF, 5 μg/mL insulin, 0.5 μg/mL hydrocortisone purchased from Promo Cell (Heidelberg, Germany), 1% anti-anti and 50 µg/mL gentamicin. The medium used for cell expansion named the Expansion Medium (Em) was composed of DMEM/F12 with 10% FBS, 5 μg/mL insulin and 0.5 μg/mL hydrocortisone (both provided by Promo Cell), 5 ng/mL hEGF (provided by Thermo Fisher) and 1% anti-anti. Transport buffer (Tb) for transepithelial electrical resistance (TEER) measurement, consisted of HBSS (Lonza) (with addition of 10 mM HEPES (VWR International) and 25 mM glucose (Thermo Fisher Scientific). The antibodies used in the different application are reported in Table 1.

### 2.2. Isolation and Culture of Porcine Mammary Epithelial Cells (pMECs)

Porcine mammary gland (MG) tissues were collected from sows (*n* = 3), namely MG2, MG3 and MG8, at a local slaughterhouse, in order to obtain three primary mammary epithelial cell lines. After collection an entire mammary line from each sow was immediately transferred to the laboratory in a temperature-controlled container (+4 °C); the tissue surfaces were firstly disinfected with Betadine (10% cutaneous solution) and ethanol 70%. The abdominal breasts were selected, transferred under a laminar flux hood than processed in aseptic conditions. Portions of mammary glands were isolated with sterile scissors and scalpels, removing cutaneous tissue, nipple and excess adipose tissues. Samples were collected for further histological and immunohistochemical analysis. The tissue was cut into pieces of approximately 1 g then minced with the scalpel into smaller pieces of 1–2 mm^3^.

The digestion of mammary tissue was performed by combining enzymatic and mechanical dissociation using the GentleMACS Octo Dissociator (Miltenyi Biotec). Mammary tissue was transferred into GentleMACS C Tubes (Miltenyi Biotec) containing the mix enzyme solution prepared following the Multi Tissue Dissociation Kit1 instructions (1 g of tissue was suspended in 3.3 mL enzymatic solution) and using the 37C_Multi_B program for 1 h. Once the run program finished, C Tubes were centrifuged at 100× *g* for 2 min to separate and subsequently eliminate the fat portion. Then, the suspension was filtered through a 100 μm and a 70 μm MACS Smart Strainers (Miltenyi Biotec). The last filter was washed with 10 mL of Im. The filtrate suspension was centrifuged at 800× *g* for 10 min, and the cellular pellet was washed once with 10 mL of Im. As indicated by [17,18] gentamicin treatment induces fibroblast apoptosis while MECs are highly resistant, therefore 50 μg/mL gentamicin was present in Im. Isolated cells were seeded in a well of a primary 6-multiwell plate in 3 mL of Im. The cells were incubated overnight at 38.5 °C in a humidified atmosphere containing 5% CO_2_. For better selection of pMECs, considering that under serum-free conditions MEC progenitors form spherical structures [19,20,21], we chose to grow cells in suspension for 24 h. Therefore, the day after, the non-adherent spheres were collected and transferred in a new well of primary 6-multiwell plate in Im added with 20% FBS. The serum allowed the spheres to adhere and cell sprouting, then the amount of FBS was reduced every 24 h, from 20% to 10% until reaching serum-free condition again to avoid growth of eventually residual fibroblasts. At ~70% confluence, cells were detached with trypsin-EDTA 1X solution, counted and seeded into a T-25 primary culture flask until 70–75% confluence was reached (first passage P). Cells were then expanded to P 10 in the Em in T25 or T75 primary culture flasks.

Cell doubling time among passages was calculated as previously described [22] for each primary cell line. For the cryopreservation, aliquots of 1 × 10^6^ cells were suspended in 1 mL of freezing medium (90% FBS and 10% DMSO). Aliquots of cell culture medium were periodically collected and analyzed by the EZ-PCR Mycoplasma Detection kit to ascertain the possible mycoplasma contamination.

### 2.3. Histological and Immunohistochemical Examination of Mammary Gland Tissues

Samples of mammary gland of 1 cm^3^ were embedded in OCT and frozen in isopentane cooled in liquid nitrogen. Seven micrometers-thick sections were cut with a Leica CM1950 cryostat (Leica, Wetzlar, Germany), then left to adhere to a microscope slide and stained with hematoxylin and eosin (H&E) according to standard procedure. Images were obtained using a Leika Aristoplan microscope equipped with a DFK 33UX264 camera (The Imaging Source Europe GmbH, Bremen, Germany). On serial sections, an immunohistochemical protocol for E-Cadherin and pan-cytokeratin (CK) was applied (Table 1). Briefly, sections were immersed in PBS, endogenous peroxidase was blocked with hydrogen peroxide 3% in methanol for 30 min at room temperature (RT). After preincubating for 30 min with 10% normal goat serum in PBS, sections were allowed to react for 1 h at room temperature with the primary antibodies (Table 1). Binding sites were revealed by secondary biotinylated antibody and amplified using an avidin–biotin–peroxidase system. Diaminobenzidine (0.04%, 10 min at room temperature) was used as the chromogen and the sections were then counterstained with Mayer’s hematoxylin for 1 min, rinsed in tap water, and mounted with glycerin jelly. In the control sections an isotype matched primary antibody of irrelevant specificity replaced the primary antibody.

### 2.4. Cell Cycle Analysis

The cells were seeded in a T25-flask and cultured until confluence, then harvested and counted. Aliquots of 8 × 10^5^ cells were washed twice in 5 mL of DPBS without calcium and magnesium and fixed overnight in 70% ice-cold ethanol (800 µL) added drop-by-drop with continuous vertexing. Then, the cells were washed with 10 mL of DPBS without calcium and magnesium and the cellular pellet was incubated with 800 µL of staining solution containing 50 µg/mL of PI and 100 µg/mL RNaseA/T1 in DPBS without calcium and magnesium for 30 min in the dark at room temperature. Cell distribution in cell cycle phases was analyzed by MACSQuant^®^ Analyzer10 (equipped with three lasers: 405, 488 and 638 nm) and Flowlogic ™ software (Inivai Technologies, Australia) as previously described [23]. FoxSynchronous Model was used to determinate the percentage of cells across the cell cycle. DNA index (DI) was calculated as the ratio between the mean G1 fluorescence level in MG2, MG3 and MG8 cells and that of the neither tumorigenic nor transformed porcine cell line, IPEC-J2 (DSMZ Leibniz Institute, Germany). A DI of 1.0 represents a normal diploid DNA content.

### 2.5. Immunofluorescence of Cytokeratins (CKs), Zonula Occludens-1 (ZO-1), Occludin (OCL)

The cells were seeded on an 8-well slide chamber (BD Falcon Bedford, Franklin Lakes, NJ, USA) at a concentration of 5 × 10^4^ cells/well. After 24 h, the cells were washed in DPBS and fixed in 4% paraformaldehyde for 15 min at RT. Subsequently, fixed cells were permeabilized with 0.5%Triton-X 100 in DPBS for 15 min at RT and then blocked with 0.5%Triton-X 100, 10% FBS in DPBS (blocking solution) for 1 h at room temperature. Then, cells were incubated ON at 4 °C in a humid atmosphere with the primary antibodies diluted in blocking solution (Table 1). Negative controls were carried out by primary antibody omission. After being rinsed in PBS (3 times 10 min each), the cells were incubated with fluorochrome-labeled secondary antisera diluted in DPBS 1 h at RT (see Table 1). After 3 washes (10 min each) in PBS, slides were counterstained by DAPI and ultimately mounted. Images were obtained using a Nikon digital camera (DS-Qi2 Monochrome Digital Microscope Camera), installed on a Nikon epifluorescence microscope Eclipse E600 and analyzed with digital image software NIS-Elements BR Ver5.30.00 (Nikon, Tokyo, Japan).

### 2.6. Epithelial Cadherin (E-Cad) and Cytokeratin18 (Ck18) Analysis by Flow Cytometry

To confirm the epithelial immunophenotype of the obtained primary cell lines, epithelial cadherin and cytokeratin 18 Flow Cytometry analysis was performed. Briefly, 3 × 10^5^ cells were fixed in 4% paraformaldehyde for 20 min at RT (300 µL) and permeabilized in absolute methanol, previously cooled at −20 °C, for 20 min at 4 °C (500 µL). Then, cells were washed in DPBS, suspended in 100 µL DPBS and incubated overnight at 4 °C in the dark with anti-E-cadherin or anti-cytokeratin 18 antibodies (Table 1). Negative controls, to evaluate inherent background or auto fluorescence, were obtained omitting primary antibodies. After incubation, cells were washed once and suspended in 300 µL of DPBS then analyzed with the MacsQuant Analyzer10. Data were analyzed using the Flowlogic™ software. To start, cellular events were discriminated from debris using forward (FSC-A) vs. side scatter (SSC-A). Doublets’ exclusion was obtained by plotting FSC-area vs height (FSC-A/FSC-H). The fluorescent staining intensity was determined comparing the median intensity fluorescence (MFI) of the negative control and the MFI of single stained cells. Positive staining was categorized as dim (slightly increased when compared to the negative control) or bright (considerable increased when compared to the negative control).

### 2.7. Barrier Study: TEER Measure and Sodium Fluorescein Transport

The monolayer formation and integrity were first assessed through the measurement of trans-epithelial electrical resistance (TEER) as previously reported for another pig epithelial cell line [24], with some important modifications. Briefly, pMECs at P10 were seeded on polyester permeable supports (pore size 0.4 µm; membrane area 0.3 cm^2^) at a density of 3.3 × 10^5^ cells/cm^2^. The transwells were incubated at 37 °C, 5% CO_2_ in Em. Medium in the apical and basal compartment was changed every 48 h. The barrier function was evaluated measuring TEER in function of time and sodium fluorescein transport every other day. Resistance (R) was measured in Tb at 37 °C, using an Epithelial Volt/Ohm Meter 2 (EVOM2, World Precision Instruments). TEER was calculated using Equation (1), where *R_total_* is the measured resistance, *R_mean_* blank is the mean resistance of the blank insert for each day and *M_area_* is the surface area of the membrane.

(1) $$TEER\left(\Omega \times {\mathrm{cm}}^{2}\right)=(R_{total}\left(\Omega \right)-R_{mean\;blank}\left(\Omega \right))\times M_{area}\left({\mathrm{cm}}^{2}\right)$$

After TEER measurements, sodium fluorescein leakage was determined. 0.5 mL sodium fluorescein (2.66 mM) and 1.2 mL of Tb, respectively, were added to the apical and basal compartment. After 1 h incubation at 37 °C under gentle shaking, samples were taken from the basal compartment. The concentration of sodium fluorescein in the samples was measured via fluorescence intensity (λ = 490/524 nm) using a Tecan Infinite M200 plate reader (Tecan Group Ltd., Männedorf, Austria).

The percentage of sodium fluorescein transport was calculated using Equation (2), where *C_sample_* is the concentration of sodium fluorescein in the sample, *C*_0_ is the donor concentration of sodium fluorescein, *V_b_* is the volume of the basal compartment and *V_a_* is the volume of the apical compartment.

(2) $$Transport\;\left(\%\right)=\frac{C_{sample}}{C_{0}}\times \frac{V_{b}}{V_{a}}\times 100$$

### 2.8. pMECs RNA Extraction and Pig Drug Transporters Analysis by RT^2^ Profiler^tm^ PCR Array

RNA extraction was performed from pMECs (0.5 × 10^6^ cells) at P10 using TRI Reagent and the NucleoSpin RNA II kit, as previously described [25]. After spectrophotometric quantification (DeNovix DS-11, DeNovix Inc., Wilmington, NC, USA) total RNA (500 ng) was reverse-transcribed to cDNA using the RT^2^ First Stand Kit. For each cell line, RT² Profiler™ PCR Array Pig Drug Transporters were performed according to the manufacturer’s instructions, using RT^2^ SYBR Green qPCR Mastermix and CFX 96 Touch (Bio-Rad Laboratories, Hercules, CA, USA). The array contained primers for 84 transepithelial transporter pathway-related genes and five housekeeping genes, 7 wells included reverse-transcription controls, positive PCR controls, and a genomic DNA contamination control. Gene expression was evaluated using the ΔCt method (mean reference genes Ct—interest gene Ct), according to the RT^2^ Profiler PCR Array Handbook.

### 2.9. Statistical Analysis

Doubling time, cell cycle, qPCR, TEER and fluorescein sodium transport data were analyzed by one-way analysis of variance (ANOVA) followed by the post hoc Tukey comparison test (*p* < 0.05) (GraphPad Prism 5 software). E-Cad and CK18 quantitative expression data by Flow Cytometry were analyzed with Student’s *t* test comparing MFI of the negative control and the MFI of single stained cells (*p* < 0.05) in each cell lines.

## 3. Results

### 3.1. Histological and Immunohistochemical Characteristics of the Mammary Glands

**Figure 1:**
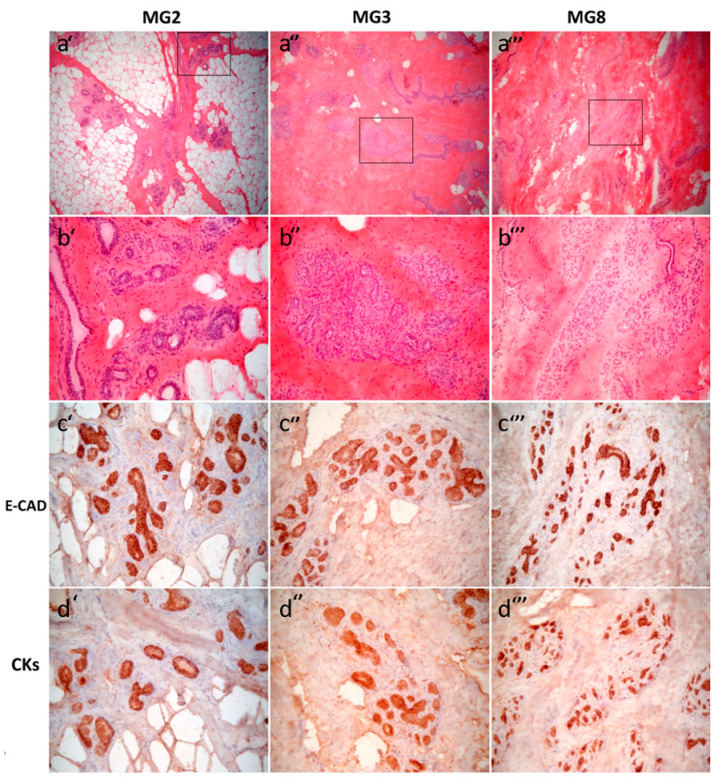
Histological and immunohistochemical features of abdominal porcine mammary glands MG2 (**a’**, **b’**, **c’**, **d’**), MG3 (**a’’**, **b’’**, **c’’**, **d’’**) and MG8 (**a’’’**, **b’’’**, **c’’’**, **d’’’**). Hematoxylin and eosin (H&E) (**a’**, **a’’, a’’’**) 6.3× objective and (**b’, b’’**, **b’’’**) 25× objective. Rectangles in (**a’, a’’**, **a’’’**) indicate areas magnified in (**b’**, **b’’, b’’’**, respectively). All three tissue samples show resting mammary gland with glandular parenchyma showing interlobular and intralobular ducts and, mainly in MG3 (**a’’, b’’**), intralobular branching ducts lacking in MG2 (**a’, b’**). There is a variable extent of adipose tissue among samples (MG2, **a’**) or dense collagen stroma (MG3 and MG8, **a’’** and **a’’’**, respectively). By immunohistochemistry, an epithelial E-Cad/panCK positive phenotype is evident in all the three MG samples (**c’**, **c’’**, **c’’’** and **d’**, **d’’**, **d’’’**, respectively).

All samples obtained from the three sows showed resting mammary gland with a mixture of adipose tissue and dense collagen stroma, embedding mammary interlobular and intralobular ducts. The latter had variable amounts of branching ducts, more abundant in sections from gilt 3 and 8 (MG3 and MG8) than in samples from gilt 2 (Figure 1). True functional alveoli with lumen containing secreted material were absent in all the samples available. Immunohistochemical results in anti-E-Cadherin-stained sections highlighted a uniform cytoplasmic staining in both interlobular and intralobular ducts, and showed diffuse staining, both cytoplasmic and, with a stronger intensity, in the plasma membrane, mainly in epithelial cells of interlobular than intralobular branching ducts.

### 3.2. pMECs Morphology, Expansion and Epithelial Marker Expression

**Figure 2:**
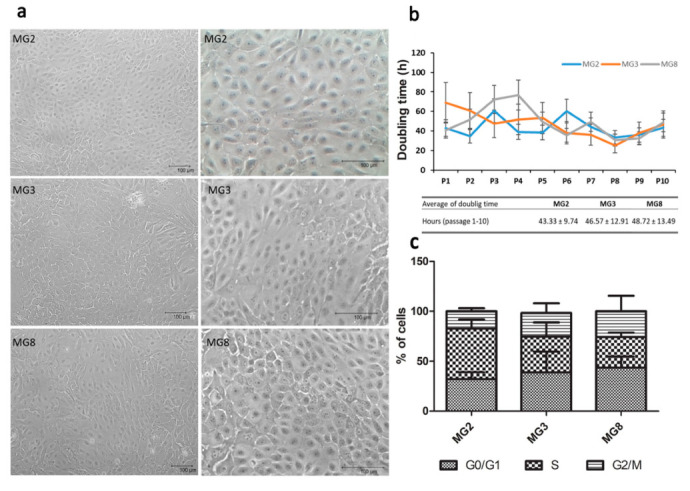
pMECs morphology, doubling time and distribution across cell cycle phases. (**a**) Representative images of cell morphology after expansion, scale bar = 100 μm; (**b**) pMEC growth curve (passage 1 to 10) for each cell line is represented, doubling time is expressed in hours, table data represent the mean ± SEM of the duplication time from P1 to P10 for each cell line. (**c**) Stacked bar chart shows cellular distribution across cell cycle phases (G0/G1, S and G2/M). Data were analyzed with a one-way analysis of variance (ANOVA) followed by the post hoc Tukey’s multiple comparison test (*p* < 0.05) (*n* = 3).

Primary cells were obtained from all three animals with different quantitative rates: 1.1 × 10^5^ cells/g of tissue from MG2 and MG8; 2.1 × 10^5^ cells/g of tissue from MG3. Cells were isolated and expanded until P10 showed a typical cobblestone morphology (Figure 2a) that was maintained through the different passages. However, in higher passages “lumen like structures” were present in all three primary cell lines, more frequently in MG8 pMECs (Figure S1). All three primary cell lines were detected as negative for mycoplasma contamination during expansion. Doubling time data indicated that cells grew with a similar rate (Figure 2b). The DNA index was normal for all three cell populations, with a mean value of 0.996 ± 0.047. Regarding cell cycle, selected single populations show the three distinct phases that could be recognized in a proliferating cell population. Statistically significant difference was not observed between the three primary lines regarding cellular distribution in the different phases of the cell cycle (Figure 2c).

**Figure 3:**
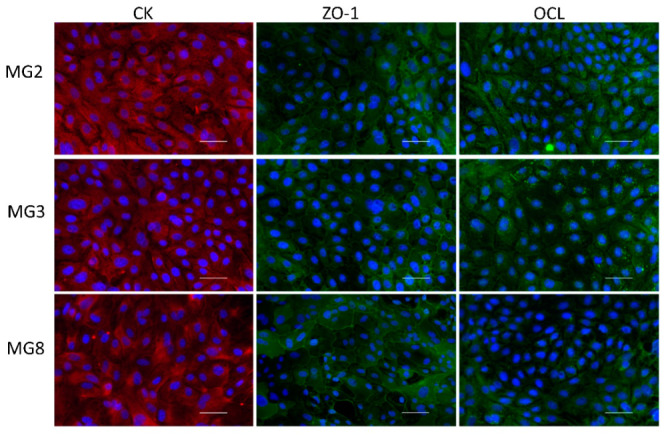
Immunofluorescence analysis of Citokeratins (CKs), Zonula Occludens-1 (ZO-1), Occludin (OCL) for MG2, MG3 and MG8 pMEC lines. Images of pMECs in the first column are representative of cells marked with the mammary epithelial CK (red); images in the other two panel columns are representative of cells marked with the tight junction proteins ZO-1 and OCL respectively (green). Nuclei are stained with DAPI (blue), scale bar = 100 µm.

Immunofluorescence analysis of typical epithelial markers indicated that pMECs from the three animals showed a diffuse positivity to the pan-cytokeratin antibody with correct cytoplasmatic localization. Moreover, immunofluorescence of the two characteristic tight junctions’ molecules ZO-1 and Occludin showed a positive signal, correctly distributed partly in the cytoplasm and partly on the membrane at intercellular junction points (Figure 3).

**Figure 4:**
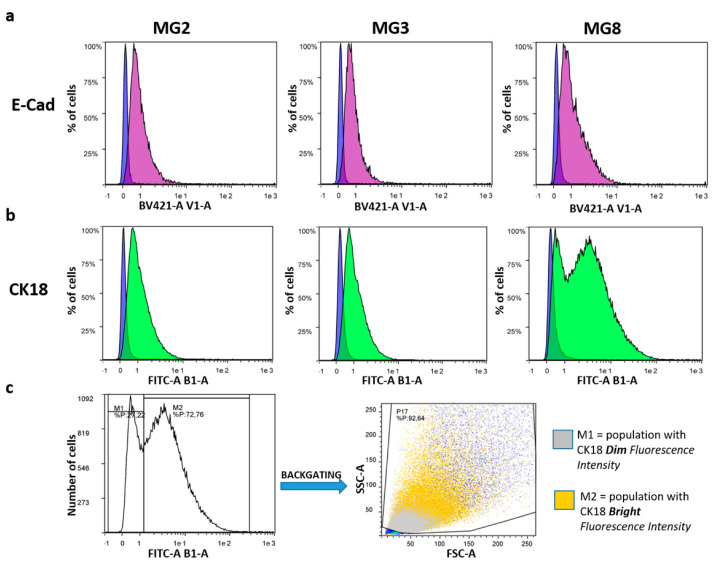
Flow cytometric analysis of porcine epithelial markers, E-cad and CK18. (**a**) Each graph shows the percentage of cells expressing E-Cad (purple Area Under the Curve—AUC) and the relative negative control (blue AUC, cells not incubated with any antibodies). (**b**) Each graph shows the percentage of cells expressing CK18 (green AUC) and the relative negative control (blue AUC, cells not incubated with any antibodies). (**c**) CK18 bimodal fluorescence peak in MG8: M1 is the fraction with a dim fluorescence intensity, and M2 the fraction with a bright fluorescence intensity. Back-gating strategy allows the visualization of M1 and M2 in the cytogram (FSC-A vs SSC-A) of MG8 cells.

Furthermore, quantitative flow cytometry analysis showed that all three cell cultures expressed epithelial markers, such as the cell adhesion protein E-Cad and the specific intermediate filament protein CK18. Specifically concerning E-Cad, the positive peaks had a dim fluorescence intensity but appeared unimodal, indicating weak positivity of the entire cellular population (Figure 4a). On the other hand, CK18 showed a dim fluorescence intensity peak in all cellular samples (Figure 4b). However, in MG8 pMECs, the fluorescence peak appeared bimodal, with one population having a dim fluorescence intensity and another one with a bright fluorescence intensity. Analyzing the peak with a back-gating strategy, it was evident that MG8 pMECs consisted of two distinct populations: one with a dim CK18 fluorescence intensity, composed of smaller and less complex cells, and the other with a bright CK18 fluorescence intensity, composed of larger and more complex cells (Figure 4c).

### 3.3. pMECs Barrier Function

**Figure 5:**
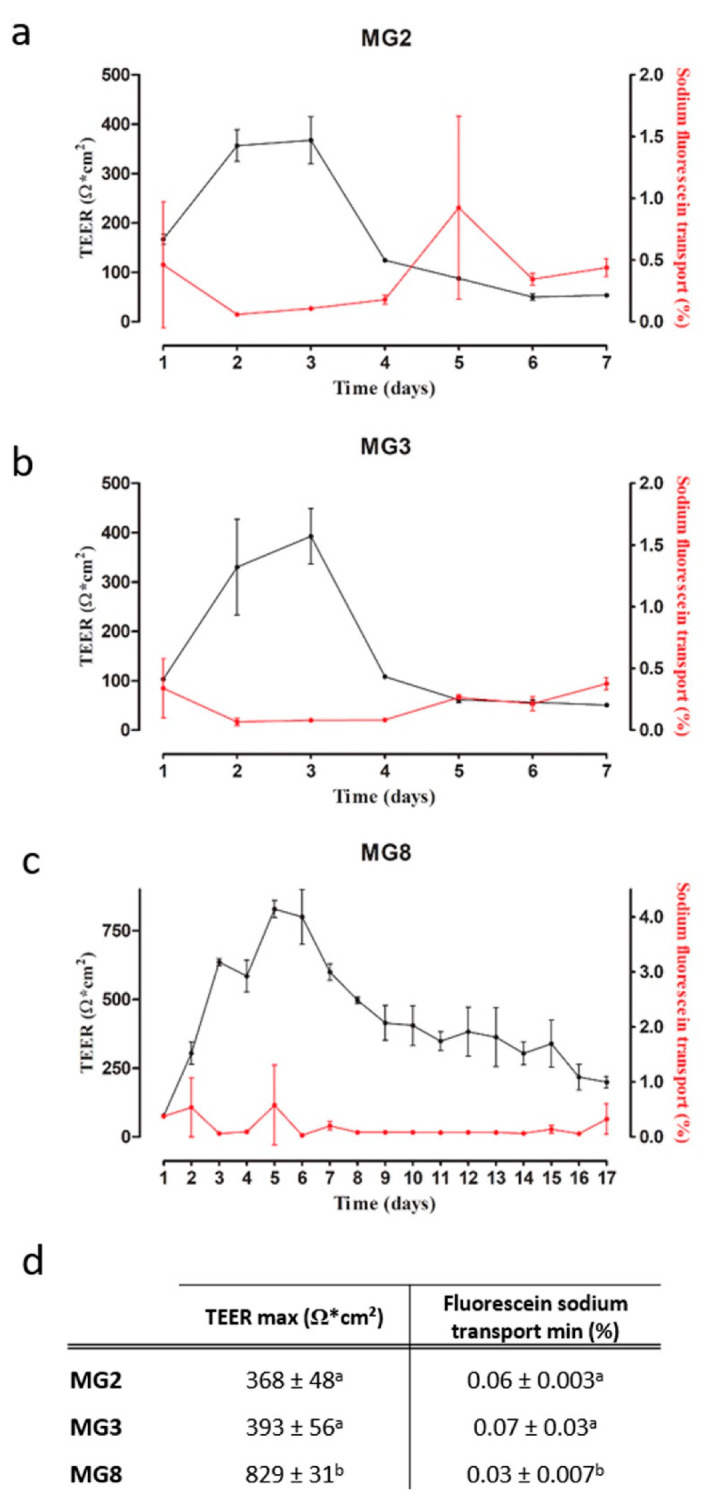
pMECs barrier function analysis via TEER and fluorescein sodium transport. (**a**–**c**) TEER (black) and sodium fluorescein transport (red) trends over the time (days) of MG2, MG3 and MG8, respectively. (**d**) TEER max and fluorescein sodium transport min data of MG2, MG3 and MG8 represented as the mean ± SD. The letters above indicate the differences between the three cell lines. Data were analyzed with a one-way analysis of variance (ANOVA) followed by the post hoc Tukey’s multiple comparison test (*p* < 0.05) (*n* = 3).

The barrier function of pMECs was evaluated via TEER and fluorescein sodium transport. TEER is a well-established method to evaluate the integrity of epithelial monolayers via measurement of the ionic conductance of the paracellular pathway [26], whereas fluorescein sodium is used to measure the passive paracellular transport [27]. For MG2 and MG3 pMECs, a plateau in TEER values (+/− 350–400 Ω × cm^2^) was already reached after two to three days, followed by a quick drop in TEER values on day four. Similarly, fluorescein sodium transport reached values around 0.06% at day two, and increased again after day four (Figure 5a,b). MG8 pMECs showed a different pattern in both TEER and fluorescein sodium transport: the initial increase in TEER was slower, a maximum of 829 Ω × cm^2^ was reached at the day 5 (Figure 5c). Afterwards, TEER values also decreased, but were still higher than the maximal values reached in MG2 (368 Ω × cm^2^) and MG3 (393 Ω × cm^2^) for up to 15 days. Similarly, fluorescein sodium transport was low in a period starting from day 3 up to day 16 (Figure 5c). Furthermore, statistically significant differences are evident in TEER maximum values and in fluorescein sodium transport minimum values reached between MG2, MG3 and MG8 (Figure 5d).

### 3.4. pMECs Drug Transporter Expression

**Figure 6:**
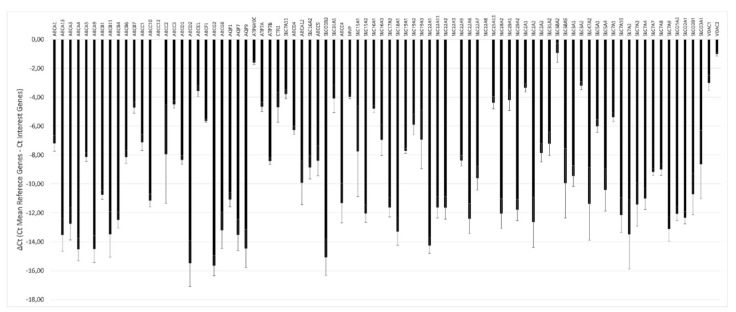
Transcriptional profile of drug transporters in the pMECs lines. Eighty-four genes of the RT^2^ Profiler™ PCR Array Pig Drug Transporters were represented. The relative gene expression was calculated as ΔCt (mean reference genes Ct- interest genes Ct) ± SD (*n* = 3).

The transcriptional profile of drug transporters showed a differential level of gene expression, ranging from ΔCt values that were very negative (lower expression) to ΔCt values that were less negative (higher expression) (Figure 6).

**Figure 7:**
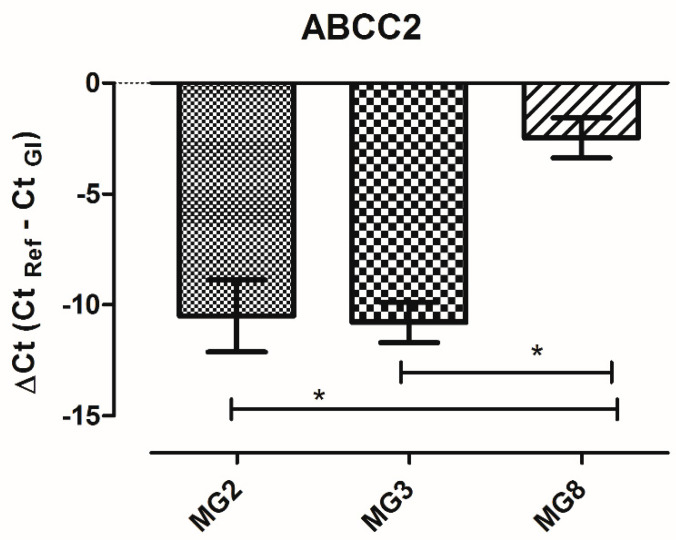
ABCC2 gene expression in pMECs data represents the mean (ΔCt) ± SD. The data were analyzed using one-way ANOVA. Significant differences are indicated by * *p* < 0.05 (*n* = 3).

Among the 84 analyzed genes, only three were not detectable (ABCC12; SLC22A3; SLC22A8). The level of expression of each gene is similar among the three animals. The only gene that shows a variation in ΔCt higher than five cycles among animals is ABCC2. A single qPCR reaction confirmed the statistically significant higher expression of ABCC2 gene in MG8 pMECs respect to the other cell lines (Figure 7).

## 4. Discussion

In the present study, we successfully developed a new method to produce pig mammary cell cultures for studying the epithelial barrier. This work is part of the IMI funded ConcePTION Project, which aims at generating further knowledge about the use of medication during pregnancy and breastfeeding, as this information is lacking for most commonly used drugs [1].

The ConcePTION consortium, taking into account multiple factors, considered the pig as the most appropriate choice of translational animal model for project purposes [3] because of physiological, anatomical and metabolic similarities with humans [2].

Currently, no companies produce primary mammary cell lines of porcine species, therefore the first goal of the present study was to develop a reproducible method of pMEC isolation and culture. To be compliant with both international legislations and the 3Rs ethical principles [28], the protocol was based on mammary tissue collected from a local slaughterhouse, which implies that the starting material could be variable. Histological investigation showed that all three tissue samples presented resting mammary glands: the secretive component (alveoli) of the gland was absent, but its potential production depends on the amount of branching ducts available [29,30]. Immunohistochemistry provided objective evidence that the epithelial component of the samples was mainly represented by epithelial cells of ducts (E-cad and pan-CK positive) instead of cells with obvious secretive phenotypes (E-cad positive but pan-CK positive associated with an intraluminal secretory component).

Regardless of tissue variability, the method that we have developed has allowed us to obtain primary cell cultures from three different animals, albeit with different quantitative percentage. The highest number of cells was obtained from MG3 and this data agrees with the indications of histology and immunohistochemistry, evidencing the highest amounts of branching intralobular ducts in this sample. The three cell line populations were successfully expanded till the 10th passage, overall data obtained from doubling time and cell cycle analysis indicated that these cells are compatible with a normal diploid proliferating population. All the three pMEC populations grew in a monolayer and exhibited typical epithelial-like morphology, in agreement with those previously obtained by Dahanayaka et al., [15] and Zheng [13].

Our results indicated that the three populations express markers typical of mammary epithelial cells: cytokeratins and epithelial cadherin. Furthermore, the flow cytometric investigation of cytokeratin 18 has allowed us to identify two subpopulations in MG8 pMECs, expressing a different intensity of cytokeratin 18. This molecule has been recognized by many authors as a specific marker for mammary epithelial cells with a highly elevated secretory profile, in many different species [21,31,32] including the pig [15]. Further investigations using final hormonal stimulation will be necessary to clarify this intriguing aspect. Overall, these results indicate that the present pMECs isolation method allows us to efficiently obtain a pure population of mammary epithelial cells. The use of gentamycin from the early stages of culture permitted the induction of fibroblast apoptosis, while MECs showed an intrinsic resistance [17,18] and the choice of culturing MECs in suspension [19,20,21] for the first day turned out to obtain the purity required.

A tight monolayer is required to evaluate transport across biological barrier including the mammary one [8]. A fundamental point supporting our model is that all three lines expressed the two important proteins Occludin and ZO-1, which make up the tight junctions that are foundational for the barrier role [33,34]. Then, the three primary cell lines obtained were tested for the ability to create a tight monolayer by TEER measurement [26]. The cells grew on PET transwell and created a tight barrier, although quantitative and kinetically different patterns existed among the three cell lines. In particular, MG2 and MG3 pMECs showed similar behavior while MG8 seemed to form a more compact barrier which remained undamaged for a longer time. These data were also confirmed by the fluorescein transport evaluation.

To our knowledge, there are no other published data relative to the TEER measurement of pMECs in vitro culture, however we can compare our results with those obtained by Kimura et al. [8] in humans. In this paper, the authors evidenced that only a selected population of trypsin-resistant hMECs were able to create a tight monolayer. It will certainly needs to be clarified if this is a species-specific difference, or is related to our method of selection that directly collected only pMECs with an intrinsic ability to form a barrier.

Another fundamental aspect is the reciprocal validation of the models obtained by integrating in vitro with the in vivo results within ConcePTION planned future activities. This will enable us to demonstrate if this intrinsic facility to form a strong barrier depends on a species-specific difference.

Finally, we have to consider that the activity of a functional barrier depends on the expression of specific transporters molecules [35]. Indeed, detailed reviews on the expression of transporters in the epithelial cells of mammary glands have been published [2,36,37,38,39]. Therefore, we characterized the drug transporter gene expression profile of the three cell lines by an array approach that includes 84 genes, belonging to the different categories of transporter families. Our results indicated that all the three lines expressed the studied genes, except the genes ABCC12 that code for multidrug resistance-associated protein 9, and the two genes SLC22A3 and SLC22A8 coding for ions transporter proteins. The most expressed genes are ABCE1, belonging to the ABC cassette family, which codes for the ATP-binding cassette sub-family E member 1, and SLC38A2, belonging to the solute carrier transporters family, which codes for sodium-dependent amino acid transporter protein.

The gene ABCC2, which codes for multidrug resistance-associated protein 2 (MRP2), was the only one with a high variation of expression among animals, showing a statistically significant higher expression in pMECs MG8, possibly in relation to sub-population complexity shown by flow cytometric investigation.

## 5. Conclusions

In conclusion, in the present paper we have reported an efficient method to obtain, from slaughtered pigs, primary mammary epithelial cells cultures as a model to study epithelial barrier function. pMECs could be utilized in pre-screening studies leading to the reduction of animals utilized for research. Overall, we believe that the results presented in this article are an excellent basis for future studies for lactational drug transfer.

## Figures and Tables

**Table 1:** Antibodies used for the immunofluorescence (IF), immunohistochemical (IHC) and flow cytometry (FC) analysis.

Antibody	P. Number	Species	Supplier	Application
Anti CK	GA053	Mouse monoclonal	Agilent Dako	IF: 1:150 IHC: 1:300
Anti E-Cadherin	610181	Mouse monoclonal	BD Transduction Laboratories	IHC: 1:2000
Anti ZO-1	61-7300	Rabbit	Thermo Fisher	IF: 1:100
Anti OCL	H-279	Rabbit	Santa Cruz Biotechnology	IF: 1:50
Anti-Rabbit IgG-Alexa Fluor 488	A11034	Goat	Thermo Fisher	IF: 5 μg/mL
Anti-Mouse IgG-Alexa Fluor 594	A11032	Goat	Thermo Fisher	IF: 5 μg/mL
Brilliant Violet 421^TM^ anti-E-cadherin	147319	Rat monoclonal	BioLegend	FC: 17 µL/10^6^
FITC anti-cytokeratin 18	ab52459	Mouse monoclonal	Abcam	FC: 10 µL/10^6^

## Data Availability

Data are available on AMSActa Institutional Research Repository by AlmaDL University of Bologna Digital Library: 10.6092/unibo/amsacta/6660.

## Supplementary Materials

The following are available online at https://www.mdpi.com/article/10.3390/ani11072012/s1, Figure S1: Representative images of “lumen like structure” in MG8 pMECs.
