# Supplementary material for: Development of a Pig Mammary Epithelial Cell Culture Model as a Non-Clinical Tool for Studying Epithelial Barrier—A Contribution from the IMI-ConcePTION Project
Source: Animals (Basel). 2021 Jul 5;11(7):2012. doi: 10.3390/ani11072012 (PMC8300391; doi:10.3390/ani11072012)
Supplement: Supplementary file 1 [file animals-11-02012-s001.zip › animals-1201470-supplementary.pdf]

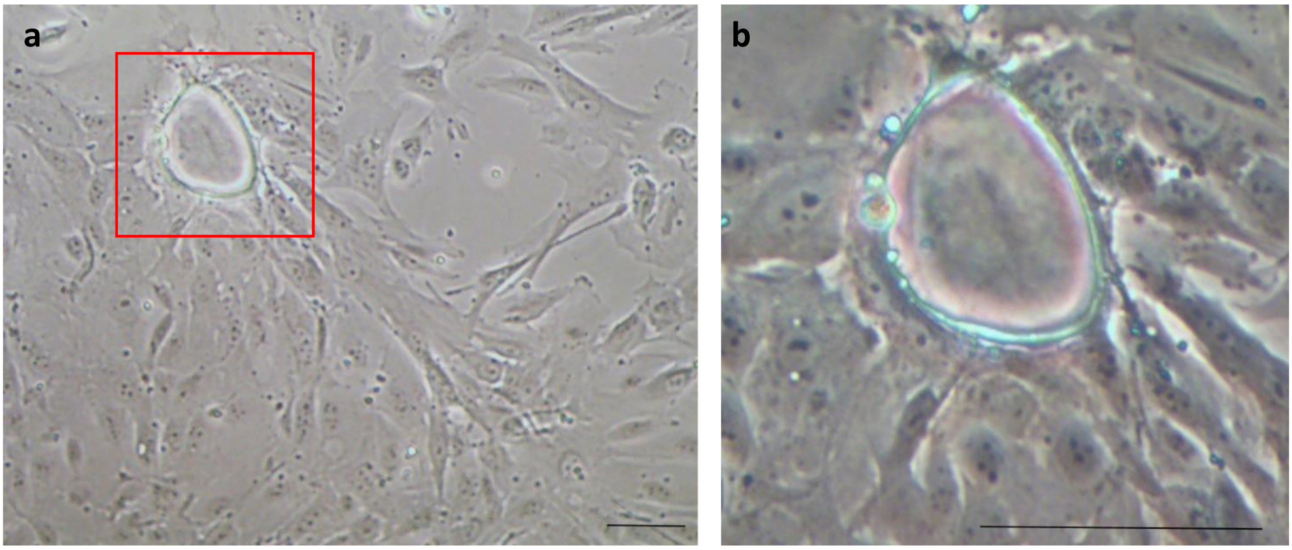

Figure S1. **Representative images of "lumen like structure" in MG8 pMECs**. Rectangle in (a) indicates area magnificated in (b). Scale bar = 100 μm;
